# Supplementary material for: Photodissociation of Cr(CO)4bpy: A Non‐Adiabatic Dynamics Investigation
Source: J Comput Chem. 2025 Jan 11;46(2):e70021. doi: 10.1002/jcc.70021 (PMC11724321; doi:10.1002/jcc.70021)
Supplement: Supplementary file 1 — Data S1 Supporting Information. [file JCC-46-0-s001.pdf]

Photodissociation of $Cr(CO)_4bpy$: A non-adiabatic dynamics investigation

Electronic Supplementary Information

Bartosz Ciborowski, Morgane Vacher

Contents

1 Franck–Condon region Benchmark **3**

2 Active spaces of CASSCF calculations **7**

3 Further benchmark on Cr-CO_{ax} PES **8**

4 Dynamic calculations **10**

List of Figures

S1 CASSCF Orbitals . . . 7

S2 PES of CAM-B3LYP Excitation Energies . . . 8

S3 PES of CASSCF(10,11)b and triplet CASSCF(6,7) methods . . . 9

S4 CASSCF(6,7) PES of an equatorial carbonyl . . . 9

S5 Bond length of all Cr–CO_{eq} . . . 10

S6 Time evolution of normal modes . . . 12

S7 Dependence of S_1/T_2 energy gap and SOCME on normal mode displacement . . . 13

List of Tables

S1 FC Benchmark of electronic structure methods . . . 4

S2 FC Benchmark of TD-DFT basis set dependence . . . 5

S3 B3LYP geometry of $Cr(CO)_4bpy$ . . . 6

S4 Contribution of $3d_{x^2}$ basis function on axial PES 8

S5 CASSCF(6,7) geometry of $Cr(CO)_4bpy$ 11

1 Franck–Condon region Benchmark

TD-DFT and CASSCF methods were benchmarked at B3LYP/6-31G* optimized geometry with a C_{2v} symmetry constraint (coordinated are present in Table S3). Results of the benchmarked along with reference data[1,2] are shown in Table S1. The functionals used are B3LYP, PBE0, BHHLYP, CAM-B3LYP. Dependence of TD-DFT excitation energies on chosen basis set is shown in Table S2. These tables are not exhaustive, as the character of high-energy TD-DFT states could not be unambiguously assigned due to their composition of many low-contribution orbital transitions, and a complete state characterisation was not the objective of this study. B3LYP and PBE0 $3d \rightarrow \pi^*_{\text{bpy}}$ excitations show charge-transfer failure,[3] manifested through underestimation of energy when compared to reference data and functionals with more, or coulomb-attenuated, exact exchange. In a reverse fashion, BHHLYP consistently overestimates $3d \rightarrow \pi^*_{\text{bpy}}$ energies by 0.2 – 0.3 eV when compared to CAM-B3LYP, while giving very similar $3d \rightarrow \pi^*_{\text{CO}}$ energies.

Though $3d \rightarrow \pi^*_{\text{CO}}$ transitions are formally of MLCT character, they do not show charge transfer failure like $3d \rightarrow \pi^*_{\text{bpy}}$ transitions do. $3d$ orbitals involved in these excitations are more precisely pairs of bonding/antibonding orbitals between chromium and neighbouring carbonyls. This can be seen in Figure S1, which showcases orbitals used in CASSCF methods, which are analogous to TD-DFT orbitals. As such, $3d \rightarrow \pi^*_{\text{CO}}$ transitions have large orbital overlap, and therefore are local in character and do not suffer from charge-transfer failure. As a consequence, CAM-B3LYP is in closest agreement with MRCI/CASSCF values, with BHHLYP being second (Mean Average Error: 0.175 and 0.337 eV).

Table S1: Transition energies of $Cr(CO)_4bpy$ at the Frank-Condon geometry. Left side contains literature data, right side has CASSCF and TD-DFT data from this work.

	Active Space:	(10,11)		(10,12)		(6,17)		(6,7)	(10,11)a	(10,11)b						
	Method:	CASSCF[a]	CASPT2[a]	CASSCF[a]	CASPT2[a]	CASSCF[b]	MRCI[b]	SA4	SA12	SA4	SA7	SA12	B3LYP	PBE0	BHHLYP	CAM-B3LYP
A_1	$d_{xz}\rightarrow\pi^*_{bpy}$	2.799	2.180			3.014	2.692	2.330	2.432	2.278	2.855	3.230	2.240	2.331	2.818	2.637
	$d_{z^2-y^2}\rightarrow\pi^*_{CO}$					3.843							3.467	3.600	3.747	3.711
	$d_{xy}\rightarrow\pi^*_{bpy}$			3.561	3.145	4.105	3.540		3.999				3.215	3.358	4.105	3.858
	$d_{xz}\rightarrow\pi^*_{bpy}$					4.166	3.809		3.751				2.919	3.077	3.936	3.623
	$d_{z^2-y^2}\rightarrow\pi^*_{CO}$					4.711									4.705	4.708
	$d_{xy}\rightarrow\pi^*_{CO}$					4.917									4.351	
	$d_{z^2-y^2}\rightarrow d_{x^2}$	5.310	4.632									5.534			5.391	4.526
	$d_{xz}\rightarrow\pi^*_{CO}$															
A_2	$d_{xy}\rightarrow\pi^*_{CO}$					3.211							3.441	3.530	3.587	3.504
	$d_{xz}\rightarrow\pi^*_{CO}$					3.313							3.151	3.201	3.264	3.158
	$d_{z^2-y^2}\rightarrow\pi^*_{bpy}$					3.943	3.339		3.801				2.850	3.043	4.082	3.703
B_1	$d_{z^2-y^2}\rightarrow\pi^*_{bpy}$	2.356	1.566			2.451	2.145	1.984	2.029	1.901	2.607	2.951	1.729	1.855	2.616	2.327
	$d_{xy}\rightarrow\pi^*_{CO}$					3.464							3.254	3.316	3.357	3.267
	$d_{z^2-y^2}\rightarrow\pi^*_{bpy}$					3.730	3.682		3.591				2.698	2.890	3.998	3.629
	$d_{z^2-y^2}\rightarrow\pi^*_{CO}$					4.209									3.746	3.998
	$d_{xy}\rightarrow d_{yz}$	4.778	3.996									4.920			5.605	5.384
	$d_{xz}\rightarrow d_{x^2}$	5.323	4.503									5.370			5.250	
	$d_{z^2-y^2}\rightarrow\pi^*_{CO}$														4.192	4.237
B_2	$d_{xy}\rightarrow\pi^*_{bpy}$	2.255	1.792			2.746	2.266	2.101	2.224	2.035	2.699	3.125	1.878	1.984	2.563	2.379
	$d_{z^2-y^2}\rightarrow\pi^*_{CO}$					3.422							2.966	3.033	3.086	3.044
	$d_{xz}\rightarrow\pi^*_{bpy}$			3.551	3.193	4.334	3.666		3.936				3.138	3.279	4.012	3.778
	$d_{xy}\rightarrow\pi^*_{bpy}$					3.951	3.843		3.740				2.833	2.996	3.801	3.651
	$d_{z^2-y^2}\rightarrow d_{yz}$	4.239	3.676								4.419	4.395	4.461		4.902	4.620
	$\pi_{bpy}\rightarrow\pi^*_{bpy}$												4.724		5.090	
	$d_{xy}\rightarrow d_{xz}$								6.006		5.018	6.046			4.208	4.396

Basis set used for DFT calculations is 6-31G*, ANO-RCC-VDZ for CASSCF, all values in eV, DFT calculations were performed on B3LYP/6-31G* geometries; [a] From Ref. [1];

[b] From Ref. [2];

Table S2: Transition energies of $Cr(CO)_4bpy$ calculated using four DFT functionals and four basis sets on B3LYP/6-31G* geometry.

		B3LYP				PBE0				BHHLYP				CAM-B3LYP			
	Transition	I	II	III	IV	I	II	III	IV	I	II	III	IV	I	II	III	IV
A_1	$d_{xz}\rightarrow\pi^*_{bpy}$	2.240	2.202	2.269	2.249	2.331	2.303	2.370	2.355	2.818	2.784	2.883	2.860	2.637	2.604	2.709	2.685
	$d_{z^2-y^2}\rightarrow\pi^*_{CO}$	3.467				3.600				3.747			3.927	3.711			3.735
	$d_{xy}\rightarrow\pi^*_{bpy}$	3.215	3.169			3.358				4.105			4.115	3.858			3.893
	$d_{xz}\rightarrow\pi^*_{bpy}$	2.919	2.870	2.939	2.908	3.077	3.037	3.103	3.078	3.936	3.726	3.769	3.746	3.623	3.588	3.660	3.631
	$d_{z^2-y^2}\rightarrow\pi^*_{CO}$									4.705				4.708			
	$d_{xy}\rightarrow\pi^*_{CO}$									4.351							4.431
	$d_{z^2-y^2}\rightarrow d_{x^2}$									5.391				4.526			
	$d_{xz}\rightarrow\pi^*_{CO}$																4.539
A_2	$d_{xy}\rightarrow\pi^*_{CO}$	3.441				3.530				3.587	3.652	3.649	3.647	3.504	3.585	3.566	3.572
	$d_{xz}\rightarrow\pi^*_{CO}$	3.151		3.178	3.180	3.201	3.244	3.228	3.229	3.264	3.314	3.303	3.303	3.158	3.215	3.198	3.203
	$d_{z^2-y^2}\rightarrow\pi^*_{bpy}$	2.850	2.824	2.911	2.882	3.043	3.027	3.111	3.088	4.082			4.090	3.703			3.757
B_1	$d_{z^2-y^2}\rightarrow\pi^*_{bpy}$	1.729	1.708	1.808	1.777	1.855	1.844	1.944	1.919	2.616	2.589	2.714	2.685	2.327	2.311	2.450	2.415
	$d_{xy}\rightarrow\pi^*_{CO}$	3.254				3.316				3.357	3.410	3.410	3.411	3.267	3.331	3.323	3.330
	$d_{z^2-y^2}\rightarrow\pi^*_{bpy}$	2.698	2.639	2.702	2.665	2.890	2.841	2.902	2.873	3.998	3.730	3.769	3.968	3.629	3.562	3.644	3.604
	$d_{z^2-y^2}\rightarrow\pi^*_{CO}$									3.746			3.753	3.998			4.034
	$d_{xy}\rightarrow d_{yz}$									5.605				5.384			
	$d_{xz}\rightarrow d_{x^2}$									5.250							
	$d_{z^2-y^2}\rightarrow\pi^*_{CO}$									4.192				4.237			4.255
B_2	$d_{xy}\rightarrow\pi^*_{bpy}$	1.878	1.857	1.961	1.939	1.984	1.975	2.076	2.060	2.563	2.544	2.663	2.641	2.379	2.363	2.500	2.476
	$d_{z^2-y^2}\rightarrow\pi^*_{CO}$	2.966	3.002	3.007	3.001	3.033	3.071	3.071	3.064	3.086	3.132	3.130	3.125	3.044	3.105	3.096	3.095
	$d_{xz}\rightarrow\pi^*_{bpy}$	3.138	3.117	3.172	3.151	3.279	3.255	3.324	3.306	4.012			4.039	3.778			3.823
	$d_{xy}\rightarrow\pi^*_{bpy}$	2.833	2.776	2.845	2.815	2.996	2.957	3.018	2.999	3.801	3.737	3.791	3.767	3.651	3.587	3.669	3.638
	$d_{z^2-y^2}\rightarrow d_{yz}$	4.461								4.902				4.620			4.612
	$\pi_{bpy}\rightarrow\pi^*_{bpy}$	4.724								5.090							4.875
	$d_{xy}\rightarrow d_{xz}$									4.208				4.396			

Basis sets used are: (I) 6-31G*; (II) 6-311G*; (III) 6-31+G*; (IV) 6-311+G*; all values in eV;

Table S3: Geometry of $Cr(CO)_4bpy$ structure optimized at the B3LYP/6-31G* level of theory, under C_{2v} symmetry constraint. All units in Å.

Atom	x	y	z
Cr	0.00000000	0.00000000	1.09152833
C	−1.90097191	0.00000000	1.17491192
C	1.90097191	0.00000000	1.17491192
C	0.00000000	1.32631596	2.36595293
C	0.00000000	−1.32631596	2.36595293
O	0.00000000	2.18497353	3.15290140
O	0.00000000	−2.18497353	3.15290140
O	−3.05294832	0.00000000	1.28580416
O	3.05294832	0.00000000	1.28580416
C	0.00000000	2.65000899	−0.49438931
C	0.00000000	−2.65000899	−0.49438931
C	0.00000000	1.51317415	−2.97647707
C	0.00000000	−1.51317415	−2.97647707
C	0.00000000	3.47898973	−1.60882677
C	0.00000000	−3.47898973	−1.60882677
H	0.00000000	3.06055570	0.50866552
H	0.00000000	−3.06055570	0.50866552
H	0.00000000	1.03902960	−3.95082853
H	0.00000000	−1.03902960	−3.95082853
H	0.00000000	4.55664385	−1.48225739
H	0.00000000	−4.55664385	−1.48225739
C	0.00000000	2.89865636	−2.87780513
C	0.00000000	−2.89865636	−2.87780513
H	0.00000000	3.51346060	−3.77216446
H	0.00000000	−3.51346060	−3.77216446
C	0.00000000	−0.73570148	−1.81258473
C	0.00000000	0.73570148	−1.81258473
N	0.00000000	1.30427946	−0.57793451
N	0.00000000	−1.30427946	−0.57793451

2 Active spaces of CASSCF calculations

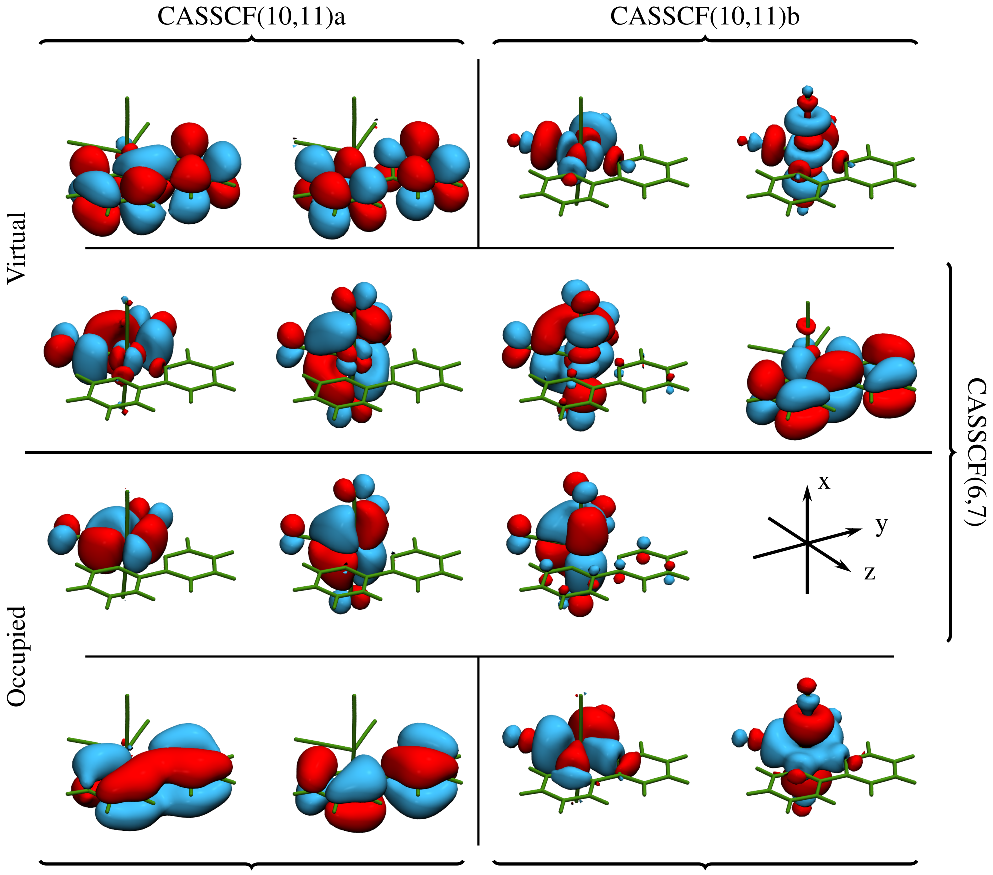

Figure S1: Orbitals of active spaces using during the study. CASSCF(6,7) is made up of the middle row of orbitals. CASSCF(10,11) (a) and (b) extend on top of CASSCF(6,7) orbitals with 2 pairs of bonding/antibonding orbitals. CASSCF(10,11)a adds orbitals on the left; CASSCF(10,11)b adds orbitals on the right.

3 Further benchmark on Cr-CO_{ax} PES

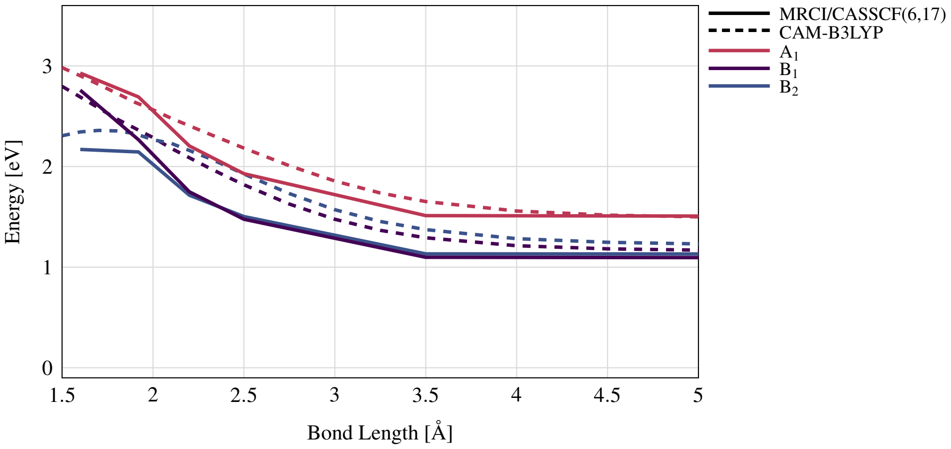

Figure S2: Excitation energies of CAM-B3LYP and MRCI/CASSCF(6,17)[2] as a function of Cr–CO_{ax} bond length.

Table S4: Contribution of the $3d_{x^2}$ basis function to the π^*_{bpy} orbital solution as a function of Cr–CO bond length.

Distance (Å)		1.903	2.1	2.4	3.0	4.0	5.0
SA4-CAS(6,7)	CO_{ax}	0.0000	0.0174	0.0409	0.0748	0.0995	0.1050
	CO_{eq}	0.0000	0.0000	0.0000	0.0000	0.0000	0.0001
SA4-CAS(10,11)b	CO_{ax}	0.0000	0.0312	0.0728	0.1548	0.3741	0.4374
SA7-CAS(10,11)b	CO_{ax}	0.0000	0.0551	0.3589	0.5417	0.5348	0.5375
SA12-CAS(10,11)b	CO_{ax}	0.0000	0.0188	0.5682	0.5421	0.5304	0.5269

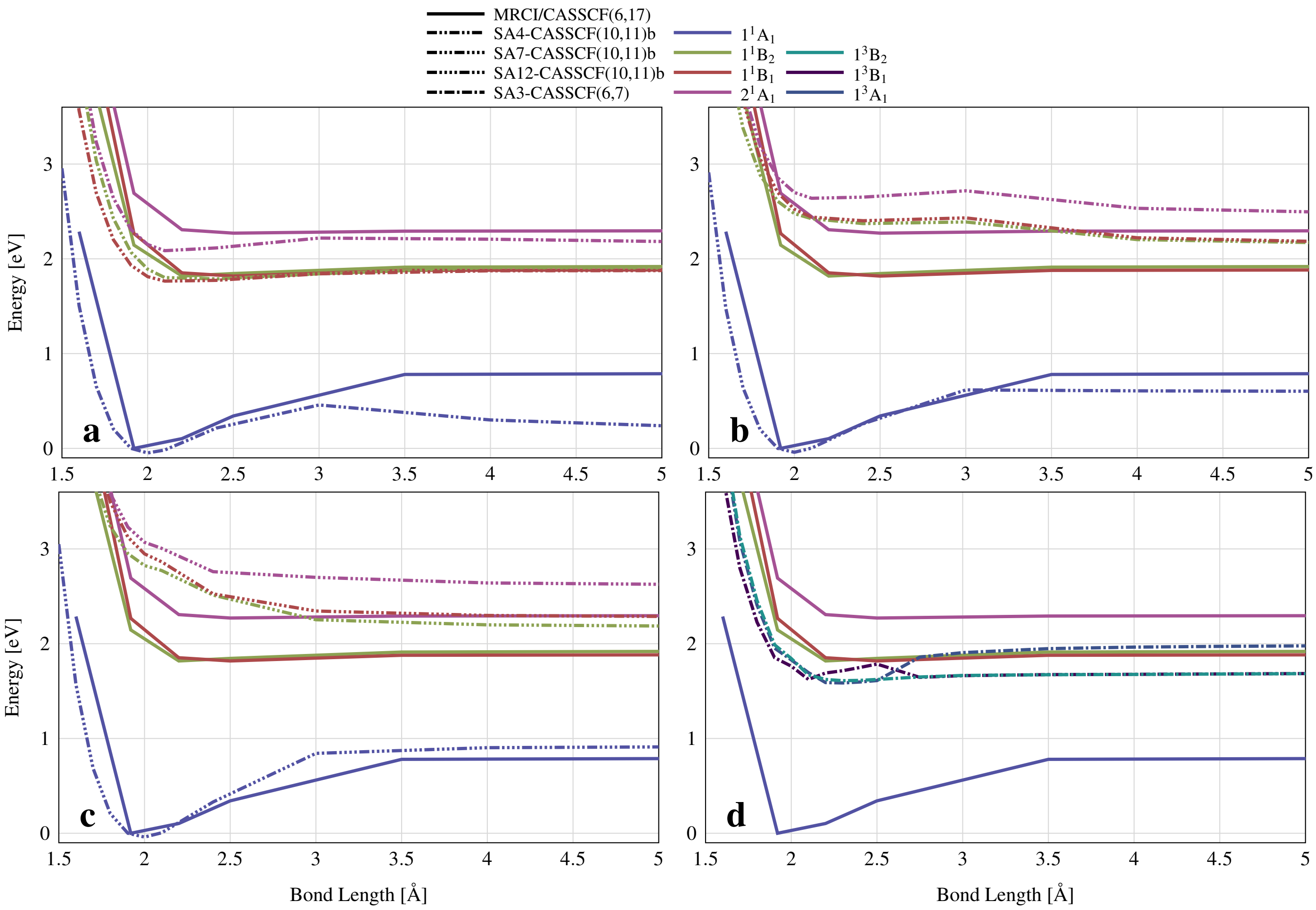

Figure S3: Potential energy surfaces of $Cr(CO)_4bpy$ as a function of Cr–CO_{ax} bond length coordinate; (**a**) SA4-CASSCF(10,11)b; (**b**) SA7-CASSCF(10,11)b; (**c**) SA12-CASSCF(10,11)b; (**d**) Triplet SA3-CASSCF(6,7); Reference MRCI data reproduced from Guillaumont *et al.*;[2] PES scans were initiated from a B3LYP/6-31G* minimum geometry; State labels correspond to the symmetry at C_{2v} FC geometry.

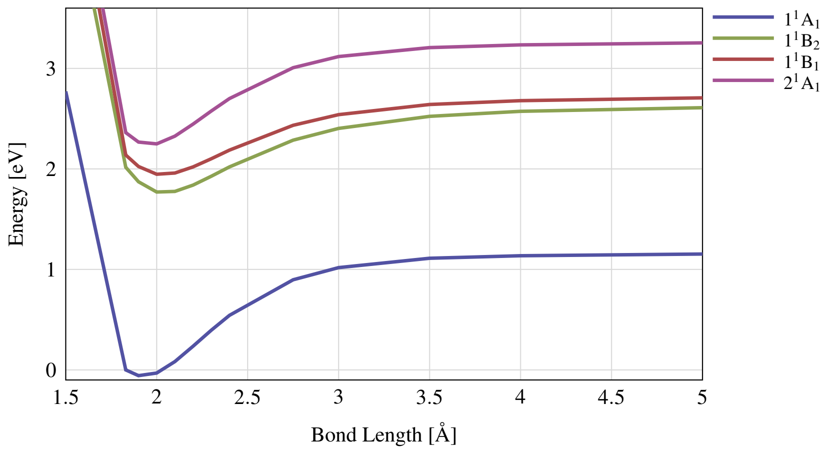

Figure S4: Potential energy surface of $Cr(CO)_4bpy$ as a function of Cr–CO_{eq} bond length coordinate using SA4-CASSCF(6,7); PES scan was initiated from a B3LYP/6-31G* minimum geometry; State labels correspond to the symmetry at C_{2v} FC geometry.

4 Dynamic calculations

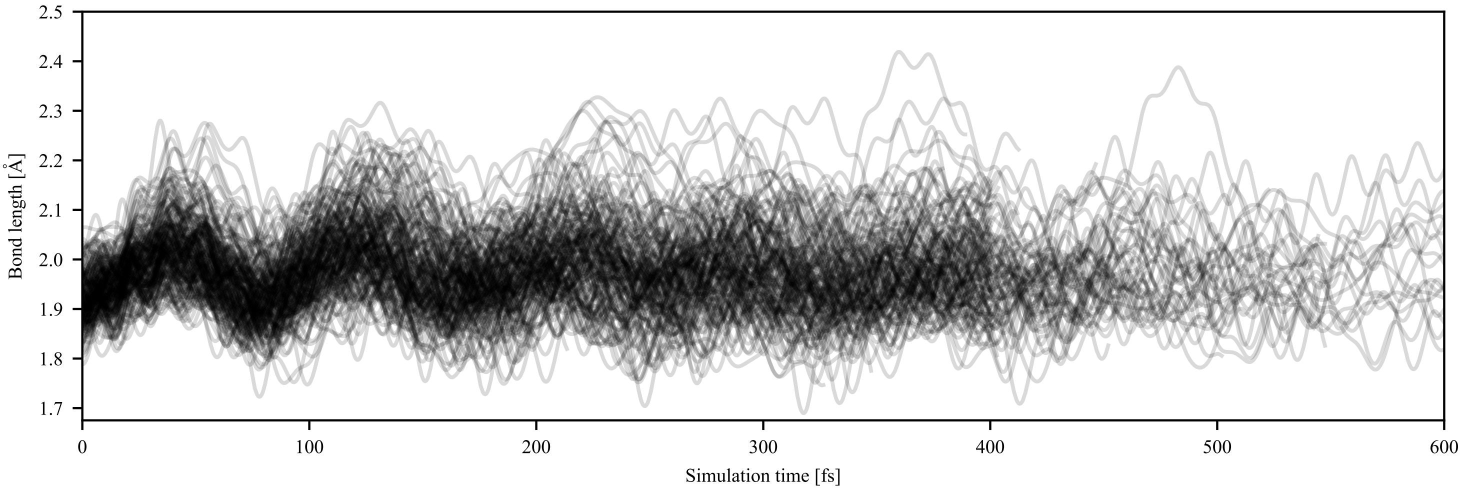

Figure S5: Evolution of the Cr–CO_{eq} bond length of all trajectories.

Table S5: Geometry of $Cr(CO)_4bpy$ structure optimized at the CASSCF(6,7)/ANO-RCC-VDZ level of theory. All units in Å.

Atom	x	y	z
Cr	0.00000325	−0.00003401	1.12178328
C	−2.00433708	0.00001346	1.18525461
C	2.00434504	−0.00004453	1.18523705
C	0.00003315	1.37877261	2.46070836
C	−0.00001155	−1.37887055	2.46066272
O	0.00003655	2.19545629	3.28051239
O	−0.00001705	−2.19558290	3.28044065
O	−3.14271240	0.00003567	1.28000614
O	3.14271990	−0.00003131	1.27999552
C	0.00000273	2.65195780	0.52822297
C	−0.00003633	−2.65190431	0.52819697
C	−0.00005912	1.50689808	2.98937965
C	0.00001557	−1.50688914	2.98936366
C	−0.00001690	3.46616136	1.63312433
C	0.00000142	−3.46613077	1.63307239
H	0.00007339	3.06615916	0.45972775
H	−0.00004311	−3.06608377	0.45976141
H	−0.00016173	1.04156094	3.95494522
H	−0.00006829	−1.04157948	3.95494203
H	−0.00010635	4.53414507	1.51215097
H	0.00000858	−4.53411163	1.51207070
C	−0.00017400	2.88189321	2.89543659
C	0.00002724	−2.88188254	2.89539706
H	0.00031138	3.48967310	3.78321785
H	0.00020371	−3.48967770	3.78316758
C	−0.00001840	−0.73785636	1.83264273
C	−0.00002009	0.73789712	1.83264291
N	0.00002062	1.30794824	0.61732767
N	−0.00002014	−1.30789313	0.61732421

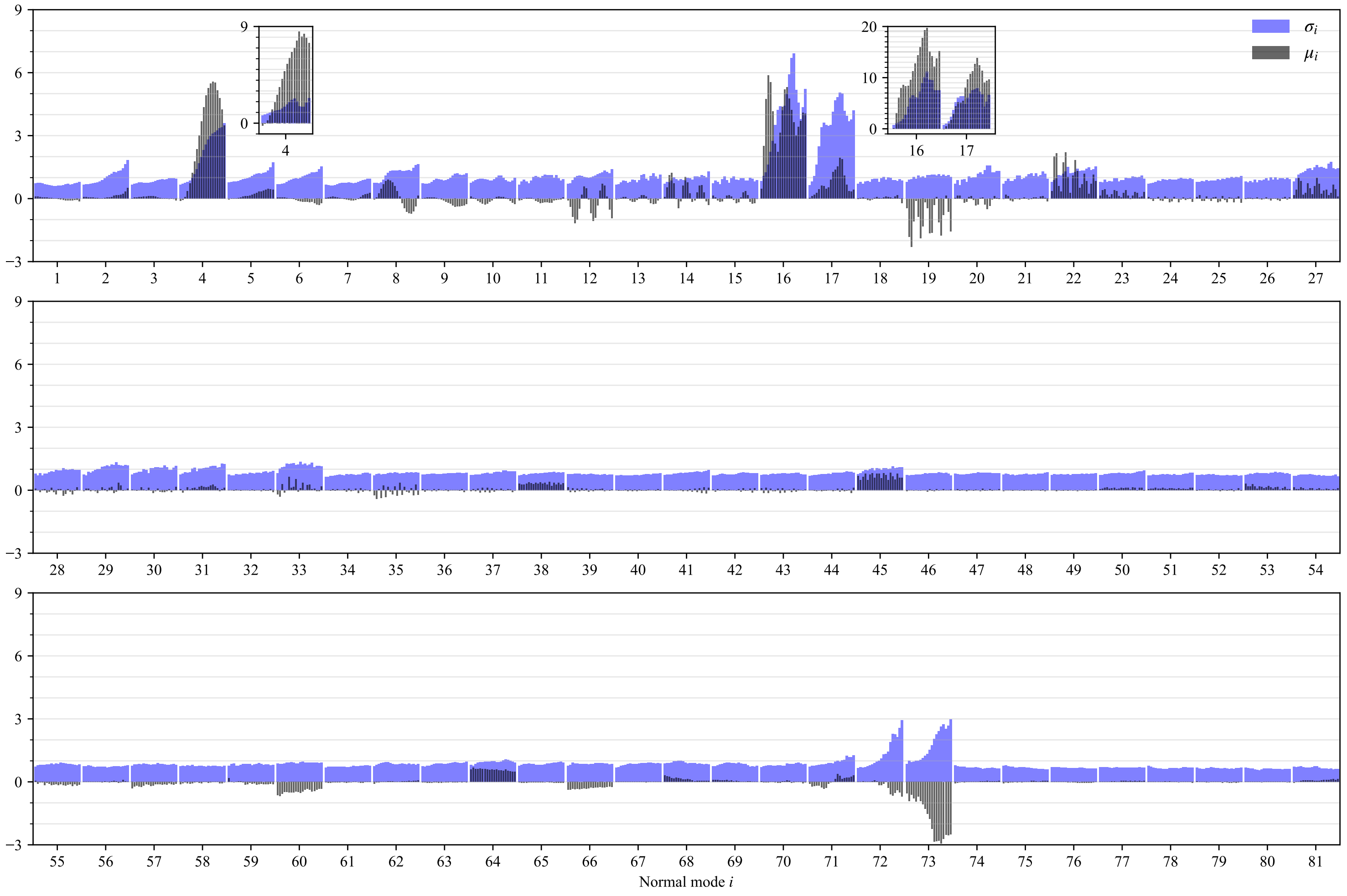

Figure S6: Mean displacement μ_i and standard deviation σ_i for each mass-frequency scaled normal mode i of the trajectory ensemble, taking the ground state minimum geometry as reference. Average values were taken over each 20 fs interval, up to the final value of 400 fs. Insets present the same analysis for a subset of dissociating trajectories, for modes 4, 16 and 17.

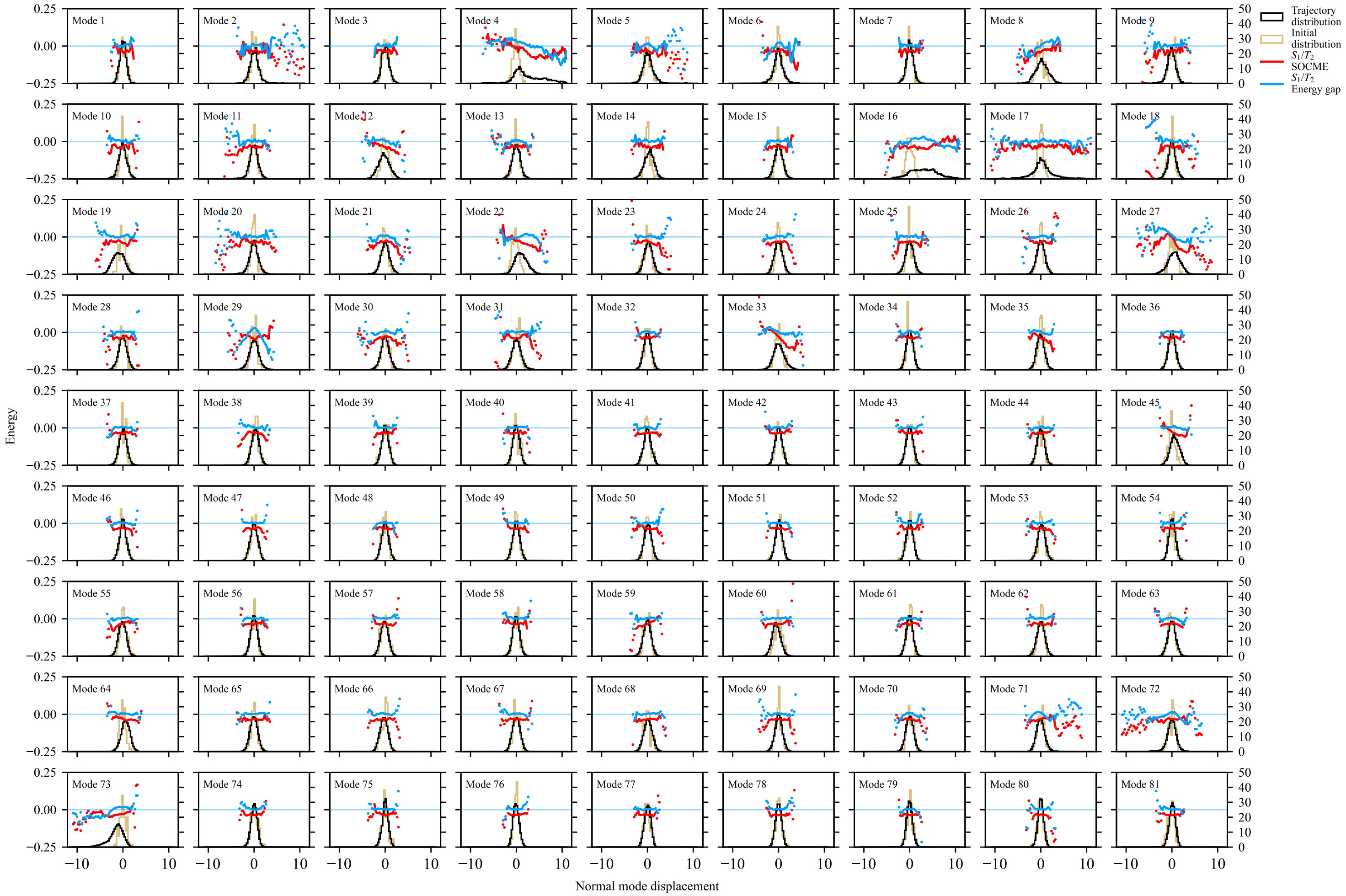

Figure S7: Energy gap and spin-orbit coupling matrix elements of S_1/T_2 crossing, averaged over the nuclear displacement for all normal modes. Distribution of all geometries visited during dynamics, and the initial Wigner distribution are given for reference. Energy gap values are given in eV (left axis), SOCME are given in cm^{-1} (right axis). Areas with under 100 samples are indicated with dots.

References

(1) D. Guillaumont, C. Daniel and A. Vlček, *Inorg. Chem.*, 1997, **36**, 1684–1688.

(2) D. Guillaumont, A. Vlček and C. Daniel, *J. Phys. Chem. A*, 2001, **105**, 1107–1114.

(3) M. J. G. Peach, P. Benfield, T. Helgaker and D. J. Tozer, *J. Chem. Phys.*, 2008, **128**.
